# Treatment period and medical care costs to achieve the first live birth by assisted reproductive technology are lower in the single embryo transfer period than in the double embryo transfer period: a retrospective analysis of women younger than 40 years of age

**DOI:** 10.1002/rmb2.12018

**Published:** 2017-02-01

**Authors:** Shunsuke Kawahara, Akane Ueda, Takashi Nakahori, Tetsuro Honda

**Affiliations:** ^1^ Department of Obstetrics and Gynecology Kurashiki Central Hospital Kurashiki Japan

**Keywords:** assisted reproductive technology, medical care costs, multiple pregnancy, single embryo transfer, treatment period

## Abstract

**Aim:**

It was examined whether the single embryo transfer policy makes the treatment period longer for couples to achieve their first live birth by assisted reproductive technology.

**Methods:**

This study retrospectively analyzed women who started assisted reproductive technology at younger than 40 years of age in the authors’ organization. The treatment periods for couples to achieve the first live birth by assisted reproductive technology, between the women who started assisted reproductive technology from 2004 to 2009 (the double embryo transfer period group, n=250), in which the double embryo transfer was predominant, and the women who started assisted reproductive technology from 2010 to 2015 (the single embryo transfer period group, n=298), in which the single embryo transfer was predominant, were compared.

**Results:**

The age at the start of assisted reproductive technology, pregnancy rate per embryo transfer, and rate of women who achieved a live birth by assisted reproductive technology per number of women who tried assisted reproductive technology were all significantly higher in the single embryo transfer period group. Among the women who achieved a live birth by assisted reproductive technology, the incidence of multiple births and severe ovarian hyperstimulation syndrome, the treatment period, and medical care costs needed to achieve the first live birth were all significantly lower in the single embryo transfer period group.

**Conclusion:**

In the single embryo transfer period group, those women who were younger than 40 years of age achieved their first live birth by assisted reproductive technology more safely, quickly, and reasonably.

## 1 Introduction

In order to avoid multiple births as a result of assisted reproductive technology (ART), single embryo transfer (SET) has prevailed during the past 15 years in Japan.^1^ In addition, in order to avoid severe ovarian hyperstimulation syndrome (OHSS) that is caused by ART, an “all freeze” strategy has been actively used during the past 15 years in Japan.^1^ The Japan Society of Obstetrics and Gynecology established guidelines that SET should be performed, but double embryo transfer (DET) can be performed in women who are ≥35 years of age or at ≥3 embryo transfer (ET) trials, but not more than two embryos can be transferred.^2^ Consequently, SET has prevailed and, in 2013, the multiple birth rate that is associated with ART was 3.6% (1488/41 051) in Japan.^3^ The multiple birth rate that was associated with ART in 2013 varied worldwide, with an incidence of <10% in Sweden, Finland, Belgium, and Quebec province (Canada); 10%‐20% in other European countries; and 28.3% in the USA.^4^ Japan is among the nations where the multiple birth rate that is associated with ART is the lowest. Although safer pregnancies have been provided with SET and “all freeze” by the avoidance of multiple births and severe OHSS, it is anticipated that the treatment period and medical care costs for couples to achieve their first live birth (LB) have increased. In this study, the treatment period and medical care costs were compared retrospectively between the DET period and the SET period in the authors’ organization.

## 2 Materials and Methods

### 2.1 Patients

As the ratio of SET/ET rose to >50% in 2010 in the authors’ organization, the women who started ART between January 2004 and December 2009 were allocated to the “DET period” group and the women who started ART between January 2010 and December 2015 were allocated to the “SET period” group. As the number of women who were ≥40 years of age was small in the DET period group, only the women who started ART before 40 years of age were analyzed.

### 2.2 Ovulation induction

In the authors’ organization, controlled ovarian hyperstimulation (COH); that is, agonist or antagonist methods, is the first choice for the women with a normal ovarian reserve. Low stimulation; that is, clomiphene with or without low doses of recombinant follicle stimulation hormone, or natural cycles, is applied on a case‐by‐case basis.

### 2.3 Counting the treatment period

Subfertile couples sometimes have an intermission in fertility treatments due to social, economic, or emotional reasons. In this study, the treatment period was counted if the treatment was performed as quickly as possible. The period of miscarriages and stillbirths was counted according to the number of gestational weeks.

### 2.4 Medical care costs

The prices for ovulation induction, oocyte pick‐up, culture of the embryos, and ET have been increasing gradually during the examined 12 years in the authors’ organization. The costs in this study are calculated with the prices and taxes in September 2016. For reference, ¥1000 was $US9.74 on September 11, 2016.

### 2.5 Statistical analysis

The data are shown as the mean±SD. The chi‐square test of independence and Welch's *t* test were used. The difference was considered to be significant at *P*<.05.

## 3 Results

The age of the women and the number of treatment cycles are shown in Table 1. The mean age was higher in the SET period group. There was no difference in the ovulation induction methods between the two groups. The ratio of performance of the “all freeze,” blastocyst transfer, and SET all were higher in the SET period group.

**Table 1:** Age of women and the number of treatment cycles in each group

Variable	The DET period group (2004‐2009)	The SET period group (2010‐2015)	*P*‐value
Number of women	250	298	–
Age (years) at the start of ART (mean ± SD)	32.7 ± 3.6	33.7 ± 3.8	<.010
Number of OPU cycles	785	541	–
Ovulation induction			
COH/low stimulation or natural, N (%)	627/158(79.9/20.1)	455/86(84.1/15.9)	NS
Number (%) of cycles, fresh ET/ “all freeze”	590/57(91.2/8.8)	314/142(68.9/31.1)	<.001
Number of embryo transfers	988	891	–
Number (%) of blastocyst transfers	575 (58.2)	692 (77.7)	<.001
Number (%) of cycles of SET/DET/TET	236/447/307 (23.8/45.2/31.0)	699/192/0 (78.5/21.5/0.0)	<.001

ART, assisted reproductive technology; COH, controlled ovarian hyperstimulation; DET, double embryo transfer; ET, embryo transfer; NS, not significant; OPU, oocyte pick‐up; SD, standard deviation; SET, single embryo transfer; TET, triple embryo transfer.

The outcomes of the treatments are shown in Table 2. Both the pregnancy rate per ET and the ratio of the women who achieved LB to the women who were undergoing ART was higher in the SET period group. Both the incidence of severe OHSS that required admission to hospital and that of multiple births were lower in the SET period group.

**Table 2:** Outcomes of the treatments in each group

Variable	The DET period group (2004‐2009)	The SET period group (2010‐2015)	*P*‐value
PR/(fresh ET+FET), N (%)	264/988 (26.7)	322/891 (36.1)	<.001
Ratio of LB/all the women who tried ART, N (%)	135/250 (54.0)	190/298 (63.8)	<.050
OHSS admissions per fresh ET, N (%)	27/590 (4.6)	6/314 (1.9)	<.050
Number of deliveries at the first live birth^a^			
Singleton/twins/triplets, N (%)	109/24/2 (80.7/17.8/1.5)	184/6/0 (96.8/3.2/0.0)	<.001

a For example, “24 twins” means 24 twin deliveries and 48 newborns. ART, assisted reproductive technology; DET, double embryo transfer; ET, embryo transfer; FET, frozen–thawed embryo transfer; OHSS, ovarian hyperstimulation syndrome; PR, pregnancy rate; SET, single embryo transfer.

Among the women who achieved a LB by ART, the mean number of the oocyte pick‐up (OPU) trials and ET trials, the treatment period, and medical care costs that were needed to achieve the first LB all were lower in the SET period group (Table 3).

**Table 3:** Mean number of treatments, period, and medical care costs to achieve the first live birth

Variable	The DET period group (2004‐2009)	The SET period group (2010‐2015)	*P*‐value
Number of OPU trials	2.4 ± 2.4	1.3 ± 1.0	<.001
Number of embryo transfer trials	3.0 ± 2.7	2.3 ± 1.6	<.010
Treatment period (months)	4.7 ± 4.9	3.4 ± 2.7	<.010
Medical care costs (¥)	830 875.0 ± 648 913.0	543 695.0 ± 260 881.0	<.001

Data are shown as the mean±SD. DET, double embryo transfer; OPU, oocyte pick‐up; SET, single embryo transfer.

## 4 Discussion

As described in the Introduction, in order to avoid multiple pregnancies and severe OHSS, SET and “all freeze” strategies have prevailed for the past 15 years in Japan. Consequently, ART has provided safer pregnancies for women and their children. As a result, it was questioned if the treatment period and medical care costs for couples to achieve their first LB by ART would have increased from the DET period to the SET period. In this study, however, it was shown that the treatment period and costs were lower in the SET period group than in the DET period group in the authors’ organization. It is supposed that this is related to the following factors: (i) the advances in blastocyst culture have decreased the loss of good embryos; (ii) the increase in the ratio of the blastocyst transfer to the whole ET has increased the pregnancy rate per ET; and (iii) the advances in vitrification technology have decreased the loss of good embryos.

There have been a few reports on the cost‐effectiveness of SET, compared to DET.^5^, ^6^, ^7^ These three reports showed that SET is more cost‐effective than DET in the sum of the medical care costs of reproduction, perinatal and neonatal treatments. This is because multiple births are more frequent in DET. In this study, although retrospective in the different periods, it was shown that SET is more cost‐effective, even if only in the reproduction treatments. If the perinatal and neonatal medical care costs are added, it is clear that SET is more cost‐effective.

**Figure 1:**
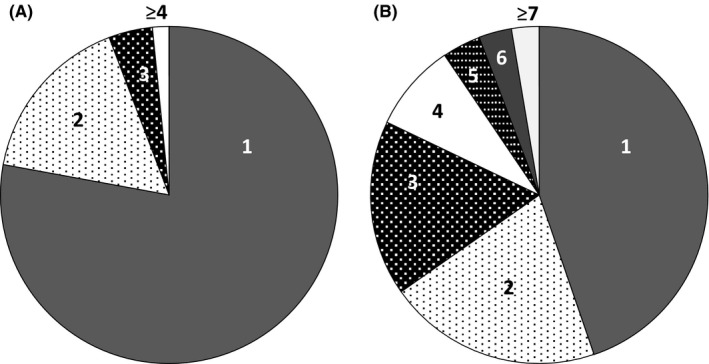
Distribution of the number of trials of (A) oocyte pick‐up and (B) embryo transfer that is needed to achieve the first live birth in the single embryo transfer period group

For declining birthrate measures, the Japanese Government provides a grant system to married couples when the wife is younger than 40 years of age and their combined annual income is <¥7 300 000: ¥300 000 for the first OPU and ET, ¥150 000 for the subsequent OPU and ET, and ¥75 000 for frozen–thawed ET, for a maximum of six times. It was shown that when women who were younger than 40 years of age underwent ART, with the first choice of COH, 63.8% of the women achieved a LB in the SET period group, and that among them, 98.4% of the women achieved their first LB within the third trial of OPU (Fig. 1A) and 97.4% of the women achieved their first LB within the sixth trial of ET (Fig. 1B). The authors believe that the Japanese grant system is well designed for young, subfertile couples.

However, even in the SET period group, there were some women who needed many trials to achieve a LB. It is speculated that, in such cases, a large number of embryos with good or fair morphology was complicated with fatal conditions; that is, aneuploidy or a genomic abnormality. The SET strategy can make it more difficult for these women to achieve a LB because their age increases before the next OPU. Pre‐implantation genetic screening (PGS) could be useful for these cases; however, at present in Japan, PGS is permitted only for couples with recurrent miscarriages. The Working Group of the Japan Society of Obstetrics and Gynecology is now reviewing the application of PGS. At present, each fertility treatment institute should create guidelines for DET for the couples to achieve a LB as quickly as possible, with the best care taken to minimize multiple births.

In conclusion, the two periods of DET and SET were compared retrospectively and showed that, in the SET period, the ratio of the women who achieved a LB increased and that the treatment period and medical care costs decreased in women who were younger than 40 years of age. In other words, ART has provided LBs more safely, more quickly, and more reasonably. It is speculated that this trend has been common during the past 15 years in Japan.

## Disclosure

Conflicts of interest: The authors declare no conflict of interest. Human rights statement and informed consent: All the followed procedures were in accordance with the ethical standards of the responsible committee of Kurashiki Central Hospital (accredited by Joint Commission International) and with the Helsinki Declaration of 1964 and its later amendments. Informed consent was obtained from all the patients who participated in the study. *Animal studies*: This article does not contain any study with animal participants that was performed by any of the authors.
